# Patient beliefs and perceptions play a crucial role in the decision-making process when managing a meniscal tear. A qualitative systematic review of the literature

**DOI:** 10.1007/s00590-021-03019-8

**Published:** 2021-05-30

**Authors:** Imran Ahmed, Fatima Dhaif, Simon G. F. Abram, Nick Parsons, Charles Hutchinson, Andrew Price, Sophie Staniszewska, Andrew Metcalfe

**Affiliations:** 1 Warwick Clinical Trials Unit, Coventry, CV4 7AL UK; 2 University Hospital Coventry and Warwickshire, Clifford Bridge Road, Coventry, CV2 2DX UK; 3 Nuffield Department of Orthopaedics, Rheumatology and Musculoskeletal Sciences, Oxford, OX3 7LD UK

**Keywords:** Meniscal tears, Patient experiences, Meta-synthesis, Qualitative studies

## Abstract

**Introduction:**

There has been an increase in research on the effectiveness of treatment options for the management of meniscal tears. However, there is very little evidence about the patient experiences of meniscal tears.

**Aim:**

To summarise the available qualitative evidence on patients’ experiences and expectations of meniscal tears.

**Method:**

A search of EMBASE, Medline, Sociofile and Web of Science up to November 2020 was performed to identify studies reporting patient experiences of meniscal tears. Studies were critically appraised using the CASP (Critical Appraisal Skills Program) checklist, and a meta-synthesis was performed to generate third-order constructs (new themes).

**Results:**

Two studies reporting semi-structured interviews from 34 participants (24 male; 10 female) were included. The mean interview length ranged from 16 to 45 min. Five themes were generated: (1) the imaging (MRI) results are a key driver in the decision-making process, (2) surgery is perceived to be the definitive and quicker approach, (3) physiotherapy and exercise is a slower approach which brought success over time, (4) patient perceptions and preferences are important in the clinical decision-making process and, (5) the impact on patient lives is a huge driver in seeking care and treatment decisions.

**Conclusion:**

This is the first study to summarise the qualitative evidence on patient experiences with meniscal tears. The themes generated demonstrate the importance of patient perceptions of MRI findings and timing of treatment success as important factors in the decision-making process. This study demonstrates the need to strengthen our understanding of patients’ experiences of meniscal tears.

**Supplementary Information:**

The online version contains supplementary material available at 10.1007/s00590-021-03019-8.

## Introduction

Meniscal tears are a common injury reported to affect 222 per 100,000 people and can be managed by non-operative or operative measures [1]. Over the last decade, research has found that the surgery may be no more effective than physiotherapy in the treatment of many of the patients with a meniscal tear [2, 3]. In response to the new evidence, treatment guidelines have been produced by the British Association for Surgery of the Knee (BASK) and the European Society of Sports Traumatology, Knee Surgery and Arthroscopy (ESSKA) [4–6]. These guidelines recommend, in the majority of cases, a period of non-operative treatment before any decisions for surgery are made. These guidelines were, however, developed following consultation with expert clinicians and scientists and did not involve patients in the decision-making process [4–6]. Previous research has highlighted the importance of treating the patient as a co-manager of their health and provide the individualized approach so that an informed treatment decision can be made [7, 8]. Patient experiences are also recognized as an important form of evidence, alongside clinical and economic evidence, in forming judgements about the effectiveness, acceptability and appropriateness of treatments [7]. Previous research in other musculoskeletal conditions has demonstrated that the use of shared decision-making aids has led to greater knowledge of the risks and benefits of a procedure and greater comfort with the decision being made [9, 10]. In addition, greater patient involvement has been shown to lead to greater patient satisfaction with the outcome of a procedure [9–11].

There has been an increase in qualitative literature in orthopedics with previous studies exploring patient experiences of hip fracture and ankle fractures [12–14]. For meniscal tears, patients were not involved in the production of recent guidelines. Research suggests patient involvement and understanding of patient experiences leads to improved patient satisfaction [9, 10]. Similar to shoulder and hip pain, patients may associate MRI findings with their symptoms even if the findings may be found in asymptomatic individuals [15, 16]. Meniscal tears have been identified in a high proportion of asymptomatic individuals [17], therefore it is important to explore the significance patients place on the MRI results. Therefore, there is a clear need to understand the patient experiences of living with a meniscal tear [18] in order to aid current treatment decision through increased awareness of the treatment pathway.

The purpose of this study is to review and summarise the evidence on patient experiences of meniscal tears and if possible generate new themes from the existing literature. The secondary purpose of this study is to explore the different qualitative methodologies used in the literature in order to plan future research.

## Methods

This review was reported in accordance with the PRISMA (Preferred Reporting Items for Systematic Reviews and Meta-Analyses) statement. The protocol was predefined and published in a peer reviewed journal. It can be found on the following reference [19].

Studies were selected for inclusion in the review based on the following eligibility criteria.

### Inclusion criteria

- Qualitative studies reporting the views and experiences of patients with a meniscal tear undergoing any treatment option (operative or non-operative).
- Qualitative studies utilizing any type of methodology were included.
- English language studies

### Exclusion criteria

- Abstract or conference publications
- Studies reporting qualitative methodology in a quantitative manner e.g., a survey reporting proportion of participants satisfied with treatment.

### Search Strategy and quality assessment

A search strategy was designed following consultation with a university librarian (see supplementary material). A search was performed on MEDLINE, Excerpta Medica Database (EMBASE), Allied and Complementary Medicine (AMED), Web of Science and Sociofile on 9th November 2020. Reference lists and gray literature were also searched to identify further citations. All citations were imported in Rayyan review software [20]. Following removal of duplicates, titles, abstracts and full texts were screened independently by two authors (IA and FD). Any disagreements were addressed by discussion with a senior author (AM or SS). Each qualitative study was independently appraised by two authors (IA and FD) using the Critical Appraisal Skills Program (CASP) for qualitative studies [21]. Any disagreements were addressed following discussion with a senior author (AM or SS) Studies were classed as ‘adequate’ if answered yes to 8–10 of the CASP criteria and ‘partially adequate’ if answered yes to 5–7 questions. This method was used in previous reviews [22]. However, leniency was used in the appraisal process as lack of reporting e.g. those sections where reviewers could not answer due to the absence of information may still generate new insights and data [23].

### Data extraction and analysis

Full texts were screened and using a data extraction proforma the following baseline data were collected: author, date, year of publication, journal, study methodology, and characteristics of patients included.

A meta-synthesis was performed to translate existing evidence into new theory [22]. This involved synthesis of the findings from the qualitative study, interpreting the results and generating new interpretations. Each included study was read and re-read by two authors (IA and FD) to produce a table of first-order constructs (direct quotes from participants) and second order constructs (author interpretation of the meaning of individual participant quotes). Any discrepancies were dealt with via discussion with a senior author (SS). Thematic coding was used to identify a cluster of themes to develop third-order constructs (review author interpretation of cluster themes).

## Results

### Screening results

**Fig. 1:**
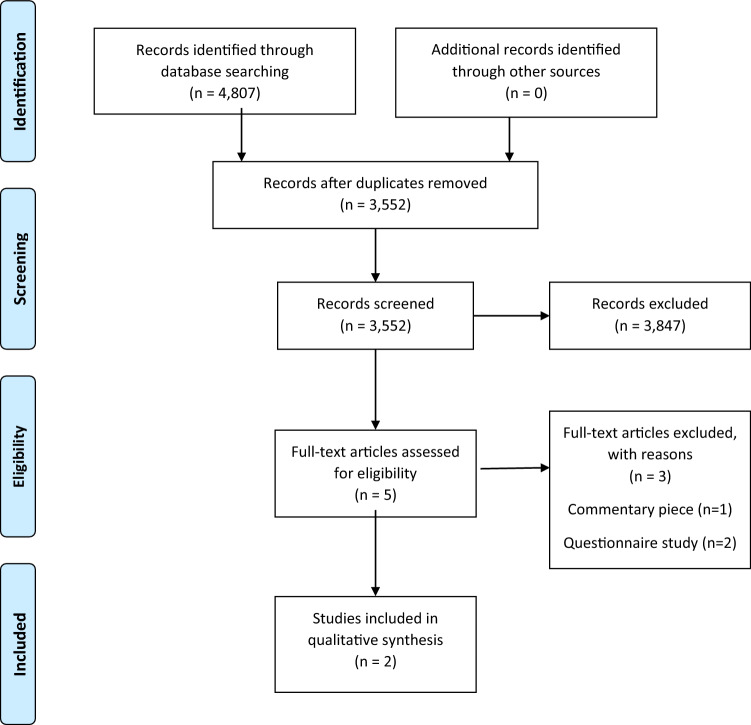
PRISMA flow diagram representing the results of the search strategy

The database search was performed on 9th November 2020, identifying 3,552 articles for screening against the eligibility criteria. After screening, five full text articles were retrieved, and two articles (two studies) were eligible for inclusion in this review[24, 25]. Reasons for exclusion included a commentary piece (*n* = 1) and questionnaire studies which were analysed in a quantitative manner (*n* = 2). Figure 1 demonstrates the PRISMA flow diagram for this review.

### Description of included studies

Two studies were included in this review, which both used semi-structured interviews in patients who presented to a secondary care setting with a MRI confirmed meniscal tear [24, 25]. Further details of baseline characteristics can be seen in Table 1.

**Table 1:** A summary of the baseline characteristics of the included studies

Study Author	Date published	Setting	Number of participants	Age	Method of diagnosis	Data collection method	Analysis method
O’Leary	2020	Secondary care in Ireland	10 (6 males and 4 females)	35–65	70% of meniscal tears diagnosed on MRI	Semi-structured interviews. Mean time 21 min (range 16 to 32)	Thematic analysis with an A-theoretical approach
Nelson	2020	Secondary care in USA	24 (19 males and 6 females)	18–50	100% of meniscal tears diagnosed on MRI	Semi-structured interviews. Range between 30 and 45 min	Thematic analysis with an A-theoretical approach

### Quality assessment

Table 2 provides a summary of the CASP grading for both studies. The only aspect of the O’Leary et al. study that did not meet the CASP standards was research design [25]. Although the research design appeared appropriate to address the aims of the study, the researchers did not discuss how and why they decided to choose semi-structured interviews and thematic approach.

**Table 2:** A table demonstrating the results of the CASP critical appraisal for each of the included studies. Studies were deemed adequate if answered ‘yes’ for eight or more questions and partially adequate if answered ‘yes’ to five to seven questions

Author	Clear statement of aims of research	Qualitative methodology appropriate?	Research design appropriate to address aims?	Recruitment strategy appropriate?	Was data collected in a way which addressed issue?	Was the relationship between researcher and participant considered?	Were ethical issues considered?	Was the data analysis rigorous?	Was there a clear statement of findings?	Will the results help locally
O'Leary 2020	Yes: To explore the benefits and treatment expectations of patients referred to secondary care with degenerative meniscal tears. Absence of evidence/ research into patient perceptions on knee pain and conservative management	Yes: Semi-structured interviews of patients attending secondary care. No information on treatment option for patients and previous exposure to treatments	Cant tell: Research design at face value is appropriate to address the aims. However, researchers did not discuss how and why they decided to choose semi-structured interviews and thematic approach	Yes: recruited from secondary care. Patients with a degenerative meniscal tear. Age range based on previous literature. Exclusion critera: did not explain why patients with OA excluded. Purposive sample taken for age/ gender/ symptoms. Not for management option and previous treatment. Explained why some patients refused to take part	Yes: Setting for data collection justified/appropriate- secondary care clinics. Semi-structured interviews clearly stated. Methods not justified. Interview schedule and topic guide available and appropriate. Data saturation discussed. However, did not specify why saturation was reached and how that decision was made	Yes: Relationship considered. Patients identified by screening waiting lists by an orthopedic physiotherapist. Clinician unknown to participants carried out interviews. Did not feature in the clinical management of patients. Interviewers had training in qualitative research. Did not specify if interviews were adapted following initial interviews etc. or whether they were all analysed in one go	Yes: Ethical issues considered. Full ethical approval granted and reference stated. Pseudonyms used. Interviews transcribed and identifying information removed	Yes: Data analysis plan documented. Thematic analysis used. Clear where themes came from as referenced to interview and quotations provided. Contradictory data not stated. Limitations clearly stated. Aware that a physiotherapist carrying out the interviewed could have led to potential bias in the findings. Acknowledged study had small study numbers and views in secondary care may not transfer to primary care	Yes: Clear discussion of findings. Strengths and weaknesses highlighted. Findings discussed in relation to original research question. Clinical and research implications discussed. Member checking was not carried out	Yes: Researchers have discussed clinical implications. Transferability and limitations with transferability to primary care. Discussed the importance of shared decision making and the more judicious use of MRI. Future research questions not clearly discussed
Nelson 2020	Yes objective and rationale clearly stated: To better understand the psychological and lifestyle factors involved in patients decisions for management of acute, non-arthritis meniscal tears	Yes: Semi-structured interviews of patients attending secondary care. Patients previous management options clearly stated	Yes: Rational for choosing semi-structured interviews clearly discussed and stated. Importance of using open-ended questions clearly stated in the methodology. Importance of interviewer training also documented clearly	Cant tell: Researcher explained how patients were selected. All patients selected at least 2 years after management decision made. Sample size determined by data saturation. No discussion of how many patients declined to participate/dropped out	Yes: Setting for data collection was justified and is appropriate. Clearly stated semi-structured interviews were used and the rational for this. Methods were justified throughout. No interview guide or schedule available. No mention on whether interviews or methods were modified. Explained how data saturation was decided. Authors are aware of the issues of reporter bias when determining data saturation	No: Although authors discussed the benefits of semi-structured interviews and open-ended questions. The authors did not discuss the relationship between themselves and interviewees. No mention on the interviewer’s role or the impact it may have on treatment	yes: Informed consent was sought. Confidentiality maintained by anonymising patient names. Ethics obtained by institutional review board	Cant tell: Data analysis plan clearly documented. Thematic analysis used. Explained reasons behind data saturation. Limitations explored. Aware of biases including social desirability bias with regards to the operative group. Face to face interviews used. No discussion of contradictory data. No analysis of their own role, potential bias	Cant tell: Clear discussion of findings. Strengths and weaknesses highlighted. Limitations included bias over data saturation and social desirability bias in the operative group. Clinical implications discussed in detail. Research implications not discussed. Used more than one analyst. No discussion of triangulation and respondent validation	Yes. Discussion of contribution to existing knowledge. Do not identify new areas where research is necessary. Do not discuss how findings can be transferred to other populations

On assessment of the Nelson et al. study, it was unclear whether the recruitment strategy was appropriate, whether the data analysis was rigorous and there was a lack of clarity regarding the statement of findings [24]. Although it was clearly stated that participants were recruited from a single center and the sample size was decided by data saturation, there was no discussion on how many participants declined to participate or dropped out. While assessing data analysis methods, the authors discussed the use of Strauss and Corbins method of coding; however, there was no discussion of contradictory data and discussion of the potential bias of the interviewers role in the study. Other limitations included that the study used more than one analyst with little description on the interactions between the analysts. There was no discussion of triangulation or respondent validation. There was also no clear discussion of the relationship and the impact of the relationship between researcher and participant.

### Meta-synthesis

Several themes were identified in the two studies which can be seen below. Table 2 demonstrates the first order (direct quotes) and second order (themes) that the study authors identified during the analysis of the interviews. The direct quotes which the themes were generated from can be seen in Table 3. The main themes identified were:

- Damaged structures cause knee problems. (Patients are) influenced by MRI findings
- Knee problems worsen over time
- The importance of the orthopedic consultation to clarify problems and lead to definitive intervention
- Surgery is a quick and straightforward solution
- Exercise is not compatible with surgery
- Perceived impairment (of meniscal tears)
- Personality influences decision making
- Time constraints influence decision making

**Table 3:** A table demonstrating the second order constructs (themes generated by authors of included studies) and the first-order constructs (direct quotes from participants)

Author	Second order constructs	First order constructs
O' Leary 2020	Damaged structures cause knee problems. Influenced by MRI findings	I'm afraid this is a cartilage problem
		I got the MRI done and it showed that the meniscus is busted. Its gone altogether
		In cases of no MRI 'I presume it’s a touch of arthritis I got'
	Knee problems worsen over time	Well its only going to get worse. What’s it going to be like in another five to ten years
		As the body gets older, naturally enough the bones and the rest of us get a bit weaker
		You need to get it done now in the short-term and save yourself long term hassle
		It getting worse and then maybe risk been off work
		Its improved an awful lot, very seldom I would get a sting off it now
		GP said give it time and it could actually go away. I was kind of saying to myself, how would it go away like, but it nearly has
	Importance of orthopedic consultation. Would clarify problem and lead to definitive intervention	So, I wanted to know what was wrong and can it be fixed
		it’s the start of a process, in the sense that, I hopefully get my knee fixed or make better than it is
		She’s the specialist, I take it she will read the MRI and tell me what it is and pursue some way to get it fixed
		I don’t want to go to orthopaedics, because I know if I go in, they would probably more than likely want to go ahead with the operation
		This referral was well over a year ago, and I have been suffering away with it ever since
		Waiting for an appointment, if I was paying private, I would have been seen when it was worse and keyhole would be done by now, but the knee is very good now to be honest
	Surgery a quick and straightforward solution	What happens is they go in, they clean it out, scrape it out or clear it up or whatever
		I’ll just go for an operation and get something done just to get it right and be able to go back to what I was used to
		Physio advised you would be better getting it sorted surgically first and then build it back up after that
		the knee is the last resort, but it doesn’t always work
	Exercise not compatible with surgery	I was a keen cyclist. When I got the results of the MRI I said I'd better stay off the bike until I get this sorted
		In my mind if I keep up cycling would I make it worse, would I do more damage than good
		Just your normal kind of recovery physio, light bending of the knee
		Exercises with the rubber band, I had to do them every morning and every night, but it just wasn’t working. Nothing was getting it right
		I was doing all the exercises. It took me 6 months to build the muscle around it. I found the physio amazing altogether
		The more exercise I did with it the better
Nelson 2020	Perceived impairment	I would worry about my knee locking on me daily
		The gym was not an option
		I wasn’t able to do anything active anymore
		The pain was so bad I wasn’t able to live my life
		Even when it wasn’t happening (something getting stuck in knee) I would always be worried about it
		I knew it wasn’t going to get better on its own
		Learned to take it easy
		learned to live with it
		PT worked well for me almost out the gate
		Pain pretty much went away completely
		Any pain I have I am able to work through
	Personality	I tend to be fairly aggressive when making decisions
		Im willing to take the risk for a definitive fix
		I am very analytical and it seemed like the highest probability for success
		I just wanted the fastest path for recovery
		I didn’t want to wast my time with PT
		It just seems like im pushing off the problem. I wanted to have a definitive treatment
		Complications can always happen
		Surgery does not always last
		I don’t believe in a perfect fix, id rather let my body heal itself
		I like to use use a watch and wait approach for anything health related
		Rather not get cut open if I don’t need to
		most things go away on their own
		From the get go I pushed myself hard in PT because I didn’t want to go through surgery
	Time constraints	I didn’t have time to go to PT first, I would have just waited a few months
		My friend told me the recovery process was quick and pretty easy
		Money was a factor for me. Work was not going to cover the time off
		The idea of being on crutches and trying to care for him definitely played on my mind
		Paying for college is stressful enough. I wasn’t sure how much my insurance was going to cover
		It would have been impossible to keep working during the recovery process

### Third-order constructs

Two authors (IA and FD) read and re-read the manuscripts and produced the following third-order constructs based on direct quotes from the interviewees available from the published manuscript.

1. *The imaging (MRI) results are a key driver in the decision-making process.*
  Participants often referred to the MRI findings as a source of the pain. One participant said the MRI demonstrated that the ‘meniscus is busted’[25], highlighting that participants believe it is an internal derangement of the knee which is the reason for the symptoms experienced. The MRI also played a vital role in the doctor-patient consultation. Participants felt that the doctor ‘will read the MRI, tell me what it is and pursue some way to get it fixed’[25]. Participants also altered lifestyle decisions and activity levels based on the MRI with one participant reporting ‘once I got the results of the MRI I said I would better stay off the bike until I get this sorted.’[25] This highlights patients’ perception of the importance of the MRI findings in determining the treatment decision.
2. *Surgery is perceived to be the definitive and quicker approach.*
  Participants believed that symptoms would not be resolved without surgery and that surgery would provide a definitive treatment. One participant felt that “the problem was not going to get better on its own” and that surgery seemed like ‘the highest probability of success’ [24]. It is for this reason one participant was ‘willing to take the risk for a definitive fix’ [24]. Patients also believed that timely surgery would be ‘able to get (patients) back to what they were used to’ [25]. It is for these reasons patients even considered ‘paying privately’ to achieve this definitive solution [25].
3. *Physiotherapy and exercise is a slower approach which bought success over time.*
  Interviews revealed a mixture of opinions regarding the success of physiotherapy and exercise for the management of meniscal tears. Patients did not ‘want to waste time with physiotherapy’[24] and felt it was ‘pushing off the problem’[24]. One patient commented that ‘if I keep cycling would I do more damage than good’[25] and despite exercising regularly one patient felt ‘it just wasn’t working’[25]. However, if patients persisted with physiotherapy many commented on the success it had on relieving symptoms. One patient explained that their symptoms ‘improved a lot, very seldom I get pain off it now’[24]. Patients also commented on the time it would take before experiencing the benefits of physiotherapy. One patient reported that ‘it took six months to build the muscles around it (the knee)’[25]. They also commented that they ‘found the physiotherapy amazing altogether’[25].
4. *Patient perceptions and decision-making preferences are an important part of the consultation and treatment journey.*
  The interviews provided a crucial insight into the treatment pathway and the importance of taking into account patient views when planning management, in particular, the amount of input patients would like in treatment decisions. Participants felt the orthopedic consultation was ‘the start of the process’ and allow the patient to get their ‘knee fixed and make it better than it is’[25]. One participant reported that the orthopedic doctor is ‘the specialist, they will read the MRI and pursue some way to get it fixed’[25]. Whereas another participant ‘wanted to know what was wrong and can it be fixed’[25]. For clinicians, it is also important to take into account patient preferences and views when planning decisions. One participant reported that they ‘are fairly aggressive when making decisions’, whereas another participant felt they were ‘highly analytical’[24]. The impact of the consultation and the recovery after surgery also had a significant impact on decision making with one participant mentioning that they ‘do not want to go to orthopedics because they would probably more than likely want to go ahead with an operation’[25]. In addition, ‘the idea of being on crutches played on the mind’ of participants when making decisions[24].
5. *The impact on patient lives is an important driver in seeking care and treatment decisions.*

A common theme amongst the interviews was the impact the symptoms of meniscal tears have on patient lives. Participants reported how they ‘would worry about their knee daily’, they ‘weren’t able to do anything active anymore’ and the ‘pain was so bad’ they were not ‘able to live their life’[24]. One patient felt it was important to ‘get it done (fixed) now in the short term and save long term hassle.’ Another believed symptoms were ‘only going to get worse’ and wondered ‘what it’s going to be like in five or ten years’[25]. These direct quotes demonstrated the huge impact symptoms had on patient lives and led to patients seeking healthcare advice and definitive management.

## Discussion

This is the first study to summarise the qualitative evidence on patient experiences with meniscal tears. The themes generated demonstrate the importance of MRI findings and patient preferences in decision making. The main finding of this review is that the experiences of patients with a meniscal tear can be grouped into the following themes: the imaging (MRI) results are a key driver in the decision-making process; surgery is perceived to be the definitive and quicker approach; physiotherapy and exercise is a slower approach which brought success over time; patient perception and decision-making preferences are an important part of the consultation and treatment journey; and the impact on patient lives is an important driver in seeking care and treatment decisions.

One of the most important findings of this review is that patient perception and decision-making preferences are an important part of the consultation. These data potentially can inform a shared decision-making process as it is important for the clinician to take into account the amount of input patients would like in the decision-making process. Greater patient involvement in the decision-making process has been shown to lead to greater satisfaction, patient knowledge of risks and benefits and comfort with the decision being made [8–11]. It is important to work with patients to determine the best course of management especially in the case of meniscal tears where there may not be one clear definitive management option [2]. It is also important to educate patients on the importance or lack of importance placed on the MRI scan. Clinicians may respond to patient expectations for a MRI, leading to an increase in MRI and surgery rates [26]. Therefore, it is important to work with patients and take into account the symptoms and examination findings before making a shared informed decision. This is in keeping with the Warwick Patient Experiences Framework which highlights the importance of viewing the patient as an active participant and taking an individualized approach in the overall patient experience [7].

The studies demonstrate that the MRI results play an important role in the decision-making process. This is in keeping with qualitative literature for other pathologies. Previous studies in hip and shoulder conditions have demonstrated that patients relate their symptoms to damaged structures on imaging even if these findings are present in asymptomatic individuals [15, 16]. Meniscal tears may be asymptomatic with up to 61% experiencing no symptoms [17]. Despite this, MRI knee is now the 2^nd^ most common MRI scan performed and MRI knees have been shown to lead to greater healthcare utilization and arthroscopy rates [27, 28]. Although MRI is a widely used investigation, this work suggests patients place a great importance on structural change, despite the literature suggesting many of these changes may not be clinically relevant. It is therefore important to clearly discuss MRI results with patients in particular which structural changes are relevant to the clinical decision-making process and which changes may not be related to patient symptoms and treatment decisions.

Surgery was perceived to be the definitive approach with many patients accepting the risks in exchange for improved outcomes. Previous reviews based on randomized trial evidence have demonstrated that, for many patients with knee pain and a meniscal tear, there may be a minimal difference in outcome between surgery and physiotherapy, with any benefit no longer present one to two years after surgery [2, 29]. The complications of knee arthroscopy are not insignificant with 0.3% of patients developing a serious complication within 90 days and 0.08% developing a pulmonary embolism [30]. Further education may be required for patients to inform them of current evidence. Patients also believed that physiotherapy was a slower approach which brought success over time, this is keeping with current trial evidence which demonstrated that physiotherapy was effective in the longer term [31]. Guidelines from Europe and the UK recommend a period of non-operative management prior to consideration for surgery for all tear types other than patients with an acutely locked knee likely secondary to a bucket handle tear [4–6].

The strengths of this study are focused around its study design. Several major databases were searched in order to identify full texts for eligibility. Two authors independently performed the search and critically appraised the included studies in detail using the CASP criteria. The methodology has been previously reported in a study on the experiences of patients with a hip fracture [22]. This is the first study to review the available qualitative literature on patients experiences with a meniscal tear. Both of the included studies contributed to the themes generated from the meta-synthesis and direct quotations were taken from each study to support these [24, 25].

The main limitation of this work is the paucity of available studies. The authors acknowledge the limitations of synthesizing the results of two studies, one of which had features of high risk of bias. However, this study highlights the evidence gap in terms of qualitative literature and patient experiences of living with a meniscal tear. In addition, there were several common themes between the two studies which the review authors believed were important to highlight to aid future clinical and research activity. Individual study designs contributed to further limitations, Nelson et al. did not discuss the relationship between the interviewer and the patient and whether the interviews role had any impact on patient management [24]. They also used more than one analyst with no discussion on triangulation and respondent validation. For both studies, the direct quotes (first order constructs) were very short. This could be due to largely closed questions asked by the interviewer. This made it difficult to generate new themes or third-order constructs from the available evidence. As there was no available interview schedule it made it difficult to further appraise the methodology. Further work is required with greater emphasis on open-ended questions in order to provide a greater insight into the patient experiences of meniscal tears. The authors of this review did not associate with a particular theoretical framework for this review. The main reason for this is due to the absence of substantial data for this subject area. The authors aimed to take a pragmatic approach and synthesize a variety of qualitative methodologies to provide a greater depth of evidence where there is a lack of studies [23].

On review of the evidence, the authors recommend that further studies are required with open-ended questions in a purposive sample of male and female patients over a range of ages undergoing both surgical and non-surgical management. It is also important to include patients that have crossed from non-surgical to surgical management given the high crossover rates previously reported in studies [29, 32]. This will not only inform future clinical practice but also the design of future large-scale trials.

## Conclusions

This study summarises the available qualitative evidence on the experiences of patients with a meniscal tear. Five themes were generated focused on the importance of imaging findings, patient experiences of operative and non-operative management, the role of patient perception and decision-making preferences on treatment decisions and the impact on patient lives is an important driver in seeking care. It is important to understand patient experiences to improve the clinical decision-making process. This study provides an insight into patient experiences of meniscal tears to add to existing clinician-derived consensus data. Further studies are required in this field to increase the amount of qualitative evidence available in order to strengthen these themes and also generate new themes.

## Supplementary Information

Below is the link to the electronic supplementary material.

Supplementary file1 (DOCX 19 kb)

## Declarations

### Availability of data and materials

The datasets used and/or analysed during the current study are available from the corresponding author on reasonable request.
