# Supplementary material for: Patient beliefs and perceptions play a crucial role in the decision-making process when managing a meniscal tear. A qualitative systematic review of the literature
Source: Eur J Orthop Surg Traumatol. 2021 May 30;32(4):619–30. doi: 10.1007/s00590-021-03019-8 (PMC9001209; doi:10.1007/s00590-021-03019-8)
Supplement: Supplementary file 1 — Supplementary file1 (DOCX 19 kb) [file 590_2021_3019_MOESM1_ESM.docx]

**Supplementary file**

**Search Strategy**

Database: Ovid MEDLINE(R) <1946 to October Week 5 2020>

Search Strategy:

--------------------------------------------------------------------------------

1 exp Tibial Meniscus Injuries/ or exp Meniscus/ (8646)

2 (menisc* adj3 (tear* or injur* or damage*)).mp. (7105)

3 1 or 2 (10614)

4 health knowledge, attitudes, practice/ or "patient acceptance of health care"/ or patient satisfaction/ (232286)

5 exp Interview/ (28579)

6 exp "Surveys and Questionnaires"/ (1051412)

7 exp Focus Groups/ (30619)

8 4 or 5 or 6 or 7 (1245380)

9 (patient$ adj3 (view$ or opinion$ or awareness or tolerance or persistenc$ or attitude$ or compliance or satisfaction or concern$ or belief$ or feeling$ or position or idea$ or preference$ or choice$)).mp. (255819)

10 (discomfort or comfort or inconvenience or bother$4 or trouble or fear$ or anxiety or anxious or worr$3).tw. (308283)

11 8 or 9 or 10 (1606707)

12 3 and 11 (762)

Database: Embase <1974 to 2020 November 09>

Search Strategy:

--------------------------------------------------------------------------------

1 exp Tibial Meniscus Injuries/ or exp Meniscus/ (15515)

2 (menisc* adj3 (tear* or injur* or damage*)).mp. [mp=title, abstract, heading word, drug trade name, original title, device manufacturer, drug manufacturer, device trade name, keyword, floating subheading word, candidate term word] (8191)

3 1 or 2 (17975)

4 health knowledge, attitudes, practice/ or "patient acceptance of healthcare"/ or patient satisfaction/ (231679)

5 exp Interview/ (294241)

6 exp "Surveys and Questionnaires"/ (736641)

7 exp Focus Groups/ (1807575)

8 4 or 5 or 6 or 7 (2827666)

9 (patient$ adj3 (view$ or opinion$ or awareness or tolerance or perception or persistenc$ or attitude$ or compliance or satisfaction or concern$ or belief$ or feeling$ or position or idea$ or preference$ or choice$)).mp. [mp=title, abstract, heading word, drug trade name, original title, device manufacturer, drug manufacturer, device trade name, keyword, floating subheading word, candidate term word] (531689)

10 (Discomfort or comfort or inconvenience or bother$4 or trouble or fear$ or anxiety or anxious or worr$3).tw. (536982)

11 8 or 9 or 10 (3511960)

12 3 and 11 (1838)

***************************

Database: AMED (Allied and Complementary Medicine) <1985 to October 2020>

Search Strategy:

--------------------------------------------------------------------------------

1 menisci tibial/ (106)

2 (menisc* adj3 (tear* or injur* or damage*)).mp. [mp=abstract, heading words, title] (177)

3 1 or 2 (231)

4 health knowledge, attitudes, practice/ or "patient acceptance of healthcare"/ or patient satisfaction.mp. [mp=abstract, heading words, title] (3528)

5 exp Interviews/ (1160)

6 interviews/ or questionnaires/ (6263)

7 Focus Groups.mp. (1062)

8 4 or 5 or 6 or 7 (10411)

9 (patient$ adj3 (view$ or opinion$ or awareness or tolerance or perception or persistenc$ or attitude$ or compliance of satisfaction or concern$ or belief$ or feeling$ or position or idea$ or preference$ or choice$)).mp. [mp=abstract, heading words, title] (3895)

10 (discomfort or comfort or inconvenience or bother$4 or trouble or fear$ or anxiety or anxious or worr$3).mp. [mp=abstract, heading words, title] (9763)

11 8 or 9 or 10 (22324)

12 3 and 11 (11)

Web of science

| # 12 | [**1,242**](https://apps.webofknowledge.com/summary.do?product=WOS&doc=1&qid=17&SID=E4sCsO5wvaXzpSQBuYm&search_mode=CombineSearches&update_back2search_link_param=yes) | #11 AND #3  *Indexes=SCI-EXPANDED, SSCI, A&HCI, CPCI-S, CPCI-SSH, ESCI Timespan=All years* | [Edit](https://apps.webofknowledge.com/WOS_AdvancedSearch_input.do?product=WOS&SID=E4sCsO5wvaXzpSQBuYm&search_mode=AdvancedSearch&replaceSetId=12&editState=init) |  |  |
| --- | --- | --- | --- | --- | --- |
|  | | | | | |
| # 11 | [**4,894,718**](https://apps.webofknowledge.com/summary.do?product=WOS&doc=1&qid=16&SID=E4sCsO5wvaXzpSQBuYm&search_mode=CombineSearches&update_back2search_link_param=yes) | #10 OR #9 OR #8  *Indexes=SCI-EXPANDED, SSCI, A&HCI, CPCI-S, CPCI-SSH, ESCI Timespan=All years* | [Edit](https://apps.webofknowledge.com/WOS_AdvancedSearch_input.do?product=WOS&SID=E4sCsO5wvaXzpSQBuYm&search_mode=AdvancedSearch&replaceSetId=11&editState=init) |  |  |
|  | | | | | |
| # 10 | [**372,330**](https://apps.webofknowledge.com/summary.do?product=WOS&doc=1&qid=15&SID=E4sCsO5wvaXzpSQBuYm&search_mode=AdvancedSearch&update_back2search_link_param=yes) | AB= (Fear OR anxiety OR comfort OR discomfort OR inconvenience)  *Indexes=SCI-EXPANDED, SSCI, A&HCI, CPCI-S, CPCI-SSH, ESCI Timespan=All years* | [Edit](https://apps.webofknowledge.com/WOS_AdvancedSearch_input.do?product=WOS&SID=E4sCsO5wvaXzpSQBuYm&search_mode=AdvancedSearch&replaceSetId=10&editState=init) |  |  |
|  | | | | | |
| # 9 | [**3,043,209**](https://apps.webofknowledge.com/summary.do?product=WOS&doc=1&qid=14&SID=E4sCsO5wvaXzpSQBuYm&search_mode=AdvancedSearch&update_back2search_link_param=yes) | AB= (Patient opinion OR views OR concerns OR perception OR attitude OR feeling OR idea)  *Indexes=SCI-EXPANDED, SSCI, A&HCI, CPCI-S, CPCI-SSH, ESCI Timespan=All years* | [Edit](https://apps.webofknowledge.com/WOS_AdvancedSearch_input.do?product=WOS&SID=E4sCsO5wvaXzpSQBuYm&search_mode=AdvancedSearch&replaceSetId=9&editState=init) |  |  |
|  | | | | | |
| # 8 | [**2,186,912**](https://apps.webofknowledge.com/summary.do?product=WOS&doc=1&qid=13&SID=E4sCsO5wvaXzpSQBuYm&search_mode=CombineSearches&update_back2search_link_param=yes) | #7 OR #6 OR #5 OR #4  *Indexes=SCI-EXPANDED, SSCI, A&HCI, CPCI-S, CPCI-SSH, ESCI Timespan=All years* | [Edit](https://apps.webofknowledge.com/WOS_AdvancedSearch_input.do?product=WOS&SID=E4sCsO5wvaXzpSQBuYm&search_mode=AdvancedSearch&replaceSetId=8&editState=init) |  |  |
|  | | | | | |
| # 7 | [**283,678**](https://apps.webofknowledge.com/summary.do?product=WOS&doc=1&qid=12&SID=E4sCsO5wvaXzpSQBuYm&search_mode=AdvancedSearch&update_back2search_link_param=yes) | AB= Focus groups  *Indexes=SCI-EXPANDED, SSCI, A&HCI, CPCI-S, CPCI-SSH, ESCI Timespan=All years* | [Edit](https://apps.webofknowledge.com/WOS_AdvancedSearch_input.do?product=WOS&SID=E4sCsO5wvaXzpSQBuYm&search_mode=AdvancedSearch&replaceSetId=7&editState=init) |  |  |
|  | | | | | |
| # 6 | [**1,496,636**](https://apps.webofknowledge.com/summary.do?product=WOS&doc=1&qid=11&SID=E4sCsO5wvaXzpSQBuYm&search_mode=AdvancedSearch&update_back2search_link_param=yes) | AB= (Surveys OR Questionnaires)  *Indexes=SCI-EXPANDED, SSCI, A&HCI, CPCI-S, CPCI-SSH, ESCI Timespan=All years* | [Edit](https://apps.webofknowledge.com/WOS_AdvancedSearch_input.do?product=WOS&SID=E4sCsO5wvaXzpSQBuYm&search_mode=AdvancedSearch&replaceSetId=6&editState=init) |  |  |
|  | | | | | |
| # 5 | [**495,449**](https://apps.webofknowledge.com/summary.do?product=WOS&doc=1&qid=10&SID=E4sCsO5wvaXzpSQBuYm&search_mode=AdvancedSearch&update_back2search_link_param=yes) | AB= Interview  *Indexes=SCI-EXPANDED, SSCI, A&HCI, CPCI-S, CPCI-SSH, ESCI Timespan=All years* | [Edit](https://apps.webofknowledge.com/WOS_AdvancedSearch_input.do?product=WOS&SID=E4sCsO5wvaXzpSQBuYm&search_mode=AdvancedSearch&replaceSetId=5&editState=init) |  |  |
|  | | | | | |
| # 4 | [**205,063**](https://apps.webofknowledge.com/summary.do?product=WOS&doc=1&qid=9&SID=E4sCsO5wvaXzpSQBuYm&search_mode=AdvancedSearch&update_back2search_link_param=yes) | AK= (Health knowledge OR attitudes OR practice OR satisfaction)  *Indexes=SCI-EXPANDED, SSCI, A&HCI, CPCI-S, CPCI-SSH, ESCI Timespan=All years* | [Edit](https://apps.webofknowledge.com/WOS_AdvancedSearch_input.do?product=WOS&SID=E4sCsO5wvaXzpSQBuYm&search_mode=AdvancedSearch&replaceSetId=4&editState=init) |  |  |
|  | | | | | |
| # 3 | [**17,293**](https://apps.webofknowledge.com/summary.do?product=WOS&doc=1&qid=5&SID=E4sCsO5wvaXzpSQBuYm&search_mode=CombineSearches&update_back2search_link_param=yes) | #2 OR #1  *Indexes=SCI-EXPANDED, SSCI, A&HCI, CPCI-S, CPCI-SSH, ESCI Timespan=All years* | [Edit](https://apps.webofknowledge.com/WOS_AdvancedSearch_input.do?product=WOS&SID=E4sCsO5wvaXzpSQBuYm&search_mode=AdvancedSearch&replaceSetId=3&editState=init) |  |  |
|  | | | | | |
| # 2 | [**17,293**](https://apps.webofknowledge.com/summary.do?product=WOS&doc=1&qid=4&SID=E4sCsO5wvaXzpSQBuYm&search_mode=AdvancedSearch&update_back2search_link_param=yes) | TS= Meniscus  *Indexes=SCI-EXPANDED, SSCI, A&HCI, CPCI-S, CPCI-SSH, ESCI Timespan=All years* | [Edit](https://apps.webofknowledge.com/WOS_AdvancedSearch_input.do?product=WOS&SID=E4sCsO5wvaXzpSQBuYm&search_mode=AdvancedSearch&replaceSetId=2&editState=init) |  |  |
|  | | | | | |
| # 1 | [**694**](https://apps.webofknowledge.com/summary.do?product=WOS&doc=1&qid=3&SID=E4sCsO5wvaXzpSQBuYm&search_mode=AdvancedSearch&update_back2search_link_param=yes) | TS= Tibial Meniscus Injuries  *Indexes=SCI-EXPANDED, SSCI, A&HCI, CPCI-S, CPCI-SSH, ESCI Timespan=All years* |  |  |  |
